# Nailfold Capillaroscopy as a Biomarker in the Evaluation of Pediatric Inflammatory Bowel Disease

**DOI:** 10.1093/crocol/otab069

**Published:** 2021-10-29

**Authors:** Jacob A Kurowski, Sonal R Patel, Joshua B Wechsler, Marisa R Izaguirre, Gabrielle A Morgan, Lauren M Pachman, Jeffrey B Brown

**Affiliations:** 1 Department of Pediatric Gastroenterology, Hepatology, and Nutrition, Cleveland Clinic, Cleveland, Ohio, USA; 2 Department of Pediatric Gastroenterology, Hepatology, and Nutrition, Ann and Robert H. Lurie Children’s Hospital of Chicago, Chicago, Illinois, USA; 3 Department of Pediatric Gastroenterology, Dell Children’s Medical Center of Central Texas, Austin, Texas, USA; 4 Department of Pediatric Rheumatology, Ann and Robert H. Lurie Children’s Hospital of Chicago, Chicago, Illinois, USA; 5 The CureJM Center of Excellence in Juvenile Myositis Research and Care, Leesburg, Virginia, USA

**Keywords:** pediatric, inflammatory bowel disease, nailfold capillaroscopy, biomarker

## Abstract

**Background:**

Noninvasive screening and disease monitoring are an unmet need in pediatric inflammatory bowel disease (IBD). Nailfold capillaroscopy (NFC) is a validated technique for microvascular surveillance in rheumatologic diseases. NFC uses magnified photography to examine nail bed capillaries called end row loops (ERL). We aimed to identify variations in NFC in pediatric IBD patients and their associations with disease activity.

**Methods:**

Pediatric patients with Crohn’s disease (CD) or ulcerative colitis (UC) and healthy controls were recruited. NFC was performed on patients with newly diagnosed IBD prior to initiating therapy, patients with established IBD, and controls. ERLs were quantified along with a 3mm distance on 8 nailfolds. Serum biomarker levels of disease activity and symptoms activity indexes were correlated with average ERL density digits on both hands. Statistics were performed using chi-squared, ANOVA, and linear regression.

**Results:**

Fifty-one IBD patients and 16 controls were recruited. ERL density was significantly decreased in IBD (Control: 19.2 ERL/3mm vs UC: 15.6 ERL/3mm vs CD: 15.4 ERL/3mm; *P* < .0001). ERL density was lower in UC patients with lower albumin levels (*P* = .02, *r*^2^ = 0.29).The change in ERL density over time predicted the change in pediatric CD activity index among CD patients (*P* = .048, *r*^2^ = 0.58) with treatment.

**Conclusions:**

Our data demonstrate ERL density is reduced in IBD compared to controls. Lower albumin levels correlated with lower ERL density in UC. In newly diagnosed CD, ERL density increases over time as disease activity improves with therapy. NFC may be a feasible biomarker of disease activity and utilized for monitoring IBD.

## Lay Summary

Nailfold capillaroscopy (NFC) is a useful technique used in other rheumatologic diseases that we examined in pediatric IBD. Our data demonstrate a reduction of nail bed capillaries called end row loops in IBD compared to controls. NFC may be a feasible biomarker of disease activity and utilized for monitoring pediatric IBD.

## Introduction

The incidence of inflammatory bowel disease (IBD) in the pediatric population has risen significantly over the past two decades. In addition to a genetic predisposition, environmental and immune system disturbances contribute to the development of chronic inflammation. Methods for screening and disease monitoring include serum and stool markers, imaging studies, and endoscopy, which can be invasive, affecting health-related quality of life. Noninvasive techniques for screening and disease monitoring can improve patient tolerance of required surveillance.

Nailfold capillaroscopy (NFC) is a technique for assessment of microvascular health in patients with juvenile dermatomyositis (JDM).^1, 2^ The pathogenesis of JDM involves an underlying microangiopathy of muscle tissue which can lead to muscle damage causing weakness.^3^ NFC utilizes high-magnification and high-resolution photographs of finger nailfolds to identify evidence of microangiopathy based on abnormal patterns in distal capillaries, termed “end row loops” (ERL). Abnormal capillary patterns in JDM include decreased ERL capillary density, capillary hemorrhage and thrombosis, areas of avascularity, and disorganization of the normal capillary patterns.^3, 4^

Vasculitis and endothelial dysfunction are contributing components of inflammation in IBD; concomitant large-vessel and antineutrophil cytoplasmic antibodies (ANCA)-associated vasculitides have been described in association with IBD.^5, 6^ Necrotizing vasculitis can occur within skin or muscle tissue in association with Crohn’s disease (CD), and leukocytoclastic vasculitis may occur even prior to the development of gastrointestinal manifestations of IBD.^7^ Additionally, vascular occlusion may occur as a result of thromboembolic events leading to gut inflammation and ischemia.^8^ Ischemia and change in the ERL capillaries in adults with active CD are similar to those in patients with systemic vasculitides.^6^

We hypothesized that NFC identifies microvascular abnormalities in IBD which distinguishes the presence of disease compared to controls and correlates with disease activity. The primary aim of this pilot study was to determine differences in ERL density between pediatric IBD patients and healthy controls. Our secondary aim was to correlate ERL density with disease activity in CD and ulcerative colitis (UC) patients using disease activity indices and serum biomarkers. The tertiary aim of this study was to correlate longitudinal changes in ERL density with changes in disease activity indices over time in newly diagnosed IBD.

## Methods

### Study Design/Patient Recruitment

We performed a prospective, longitudinal, case-control study from 2015 through 2017 of IBD patients and healthy controls. IBD patients were recruited from a subspecialty clinic at Ann and Robert H. Lurie Children’s Hospital of Chicago, inpatient wards, and prior to endoscopy. Patients 3–20 years of age were included if they had an established diagnosis of CD or UC. Confirmation of disease was determined through a review of endoscopic and histologic reports. Patients with comorbidities including eosinophilic gastrointestinal disorders, celiac disease, liver disease, cardiac disease, connective tissue disorders, rheumatologic disorders, and endocrine disorders were excluded. Patients with IBD-unclassified, self-limited/infectious colitis, or colitis related to immunodeficiency were also excluded. Healthy controls with no family history of IBD were recruited from urban, primary care clinics associated with Ann and Robert H. Lurie Children’s Hospital of Chicago. Because the process of NFC requires patients to remain still during photography, patients deemed unable to sit for or tolerate NFC were excluded from participation. Patient nailbeds were also inspected prior to recruitment and excluded if significant damage (biting, injury, etc). All patients were consented/assented, after obtaining age-appropriate consent as outlined in the approved proposals (IRB# 2014-15582) from the Institutional Review Board of Ann and Robert H. Lurie Children’s Hospital of Chicago.

### Data Collection

Patients completed a questionnaire regarding demographics, IBD history including disease type, duration, location, extra-intestinal manifestations, surgical and family history, and previous and current medications. The pediatric CD activity index (PCDAI) or pediatric ulcerative colitis activity index (PUCAI) was recorded for each patient based on disease type.^9^ Laboratory data obtained within 2 months of NFC measurements per the standard of care included serum inflammatory markers (erythrocyte sedimentation rate (ESR)/c-reactive protein (CRP)) and albumin. Chart review was performed to confirm IBD diagnosis and to identify potential exclusion criteria based on medical and surgical histories.

### Longitudinal Population

A subset of patients were newly diagnosed cases of IBD and recruited prior to the initiation of therapy. These patients unde rwent study of their NFC during multiple time points at various intervals from 2 weeks to 6 months after initiation of treatment.

### Nailfold Capillaroscopy

NFC was performed on digits # 2–5 bilaterally, excluding the thumbs of both hands, for a total of 8 nailfold measurement as previously reported.^1, 2^ The procedure was performed in a manner similar to the rheumatologic utilization of NFC as described.^4^ A Sony Cyber-Shot DSC-W710 digital camera equipped with a Dermlite II Pro Dermatoscope was used for photography of nailfolds. The Sony Cyber-Shot camera provided 5 times optical zoom and the Dermlite II Pro Dermatoscope provided 10 times optical zoom (total 50 times optical zoom). Mineral oil was applied to the distal nailfold to enhance image quality, and photographs of the end row capillary loops were obtained. Photographs were analyzed using Adobe Photoshop Creative Cloud 2015 with digital photographs scored by 2 trained reviewers (J.A.K., J.B.B.) after training by an expert in NFC (G.A.M.), which we have previously shown to be reliable when using this method.^10^ The reviewers were blinded to the disease status of the patient at the time of the scoring. A scaled 3mm ruler was inserted into each photograph to determine the density of ERLs along a 3mm distance. A previously established method for counting ERLs has been described by Hofstee et al and was used as the standard for quantification in our study.^11^ This method entails inclusion and quantification of a capillary loop in the end row of capillaries if the angle between the adjacent capillaries is greater than 90degrees. The mean ERL density along the 3mm distance was calculated for digits 2–5 on both hands, converted to mean ERL density/mm, and utilized for subsequent analysis.

### Statistical Analysis

Demographic data were analyzed using Fisher’s exact test. Linear regression analysis and 1-way ANOVA testing was used for the analysis of mean ERL density. GraphPad Prism version 6.0 was utilized for all statistical analyses. A *P*-value of less than .05 was considered to be statistically significant.

## Results

### Patient Demographics

Fifty-one IBD patients (34 CD and 17 UC) and 16 healthy controls underwent NFC assessment. The demographic features of the study population are shown in Table 1. The mean ± SD age of the cohort at the time of study entry was 11.8 ± 4.3 years. The IBD population was older than the control population (CD: 12.4 ± 4.5, UC: 12.9 ± 3.4, Control: 9.8, *P* = .064). There were no differences in gender or race between the CD, UC, and control groups. A greater percentage of patients in the control group were of Hispanic ethnicity compared to the IBD population (CD: 0%, UC: 24%, Control: 50%, *P* < .0001).

**Table 1.** Demographics

Demographic	Control (*N* = 16)	UC (*N* = 17)	CD (*N* = 34)	*P*-value^a^
Age (years), median (±IQR)^a^	9.8 ± 4.2	12.9 ± 3.4	12.4 ± 4.5	.064
Male, *n* (%)	10 (63)	8 (47)	18 (53)	.67
Race, *n* (% Caucasian)	14 (88)	11 (65)	24 (71)	.3
Ethnicity, *n* (% Hispanic)	8 (50)	4 (24)	0 (0)	**<.0001**

Abbreviations: CD, Crohn’s disease; IQR, interquartile range; UC, ulcerative colitis.

Chi-squared test of homogeneity, *P* < .05 considered significant in bold.

### Comparison of ERL Density in CD, UC, and Control Groups

**Figure 1.**
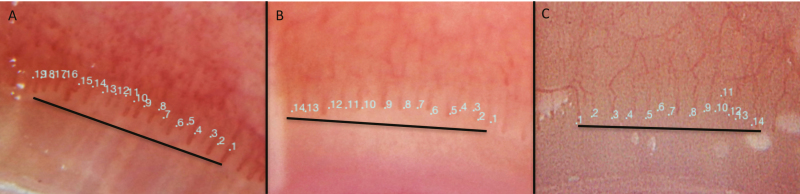
Representative photographs of nailfold capillaroscopy with 50× magnification illustrating ERL density per 3mm distance in (A) control, (B) ulcerative colitis, and (C) Crohn’s disease.

**Figure 2.**
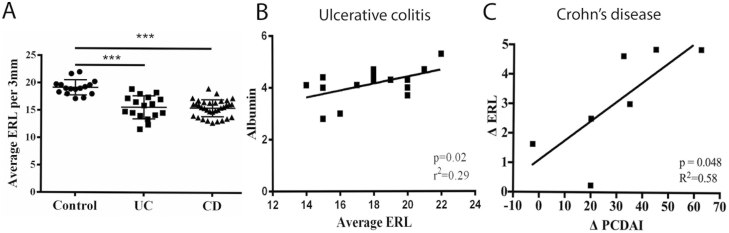
(A) ERL density is reduced in children with inflammatory bowel disease. Average ERL density from digits 2 to 5 from both hands was compared between non-IBD controls and IBD patients with either CD or UC. Comparison by non-parametric Kruskal–Wallis test with Dunn’s post-hoc test (∗∗∗*P* < .001). (B) Correlation of mean ERL density of digits 2–5 on both hands with albumin was appreciated in patients with UC. (C) The change in ERL density with treatment correlates with the change in symptom scores in children with CD. The change (delta) in average ERL density among digits 2–5 on both hands before and after treatment was correlated to the change in the pediatric Crohn’s disease activity index (PCDAI).

To determine if there was a difference between ERL density in IBD patients compared to healthy controls, we calculated the average ERL density for digits 2–5 for both hands (Figure 1). The ERL density was compared between patients in the control, UC, and CD groups. ERL density was reduced in IBD patients compared to controls with a median of 19.0 [interquartile range (IQR) 18.2, 19.7] ERL/3mm in control patients, 15.9 [14.1, 17.3] ERL/3mm in UC patients, and 15.6 [14.0, 16.5] ERL/3mm in CD patients (*P* < .001) (Figure 2A). There was no significant difference in mean ERL density between the CD and UC groups. There was also no significant difference in ERL density when comparing age, gender, and race.

### Correlation of ERL Density to Biomarkers of Disease Activity

To determine the utility of NFC as a measure of biochemical disease activity in IBD, mean ERL density in CD and UC populations were correlated to serum inflammatory markers as well as disease activity indices. In CD patients, no significant correlation was observed between mean ERL density and ESR, CRP, albumin, and PCDAI. In UC patients, a moderate correlation between mean ERL density and albumin was appreciated (*r*^2^ = 0.29, *P* = .02, Figure 2B). In UC patients, a significant association was not observed between ERL density and the following: ESR, CRP, and PUCAI, although there was a trend toward increased mean ERL density with decreasing CRP (*P* = .2).

### Longitudinal Patients

To determine the utility of NFC to predict the change in symptoms with treatment, we assessed the relationship of ERL density to PCDAI over time. Ten CD patients were recruited at the time of disease diagnosis during inpatient hospitalization. Seven of these patients were followed longitudinally. After initiating therapy, the median [IQR] PCDAI improved from 47.5 [35.6, 53.1] to 17.5 [11.3, 23.8]. The median [IQR] ERL density increased from 15.7 [14.7, 16.3] ERL/3mm to 18.4 [18.0, 19.1] ERL/3mm. The change in ERL density was associated with change in PCDAI (*r*^2^ = 0.58, *P* = .047, Figure 2C) at 6-month follow-up utilizing linear regression.

## Discussion

In this study, we found average ERL density was decreased in IBD patients compared to healthy controls and ERL density correlated with albumin in patients with UC. Previous investigation by Gasser et al in adults found average ERL density of 5.5/mm in CD patients compared to 7.1/mm in controls (*P* = .001), similar to our findings of 5.2/mm in pediatric CD and 6.2/mm in pediatric controls (*P* < .001).^6^ This identifies a potential role for NFC as a noninvasive marker to identify and monitor IBD, warranting larger studies.

To date, there are few studies investigating the relationship between microangiopathy and IBD since it was first described in association with UC in 1949.^5^ The majority of work has evaluated the intestinal histology or intestinal resections using histologic evaluation. Kruschewski and Buhr published the largest series examining the correlative relationship of IBD and vasculitis.^12^ They detected vasculitis using immunohistologic staining of endothelin-1 in 95% (*n* = 39/41) of colonic specimens with moderate to severe histologic inflammation compared to quiescent or mild inflammation in both UC and CD. Saijo et al reported vascular permeability in the distal colon increases after dextran sulfate sodium (DSS) administration in a mouse model.^13^ Furthermore, vascular wall injury occurs in the lamina propria after DSS administration leading to colonic epithelial damage. In JDM, it is previously described that decreased ERL density is associated with a decrease in the bioavailability of enteral steroids.^14^ It is possible that these vascular changes play a role in the pathophysiology and treatment response of IBD. We did not find other changes as seen with JDM including capillary hemorrhage, tortuosity, or dilation. Hypoalbuminemia is a well-studied marker of disease severity and predicts the need for a higher initial dose of infliximab in fulminant UC, presumably due to vascular leakage of anti-TNF antibody.^15^ In our study, ERL density did not correlate with albumin and measures of disease activity in CD. The biochemical disease assessments (ESR, CRP) are representative of acute active disease that quickly changes with treatment. Similar to JDM, we hypothesize that changes to NFC in IBD are indicative of the chronic disease process that takes months to appear and resolve. However, a significant correlation was found with albumin in UC. Whether this decrease in both albumin and ERL density indicates a component of vascular leak in the setting of vasculitis is unclear. It may be possible to utilize NFC as a biomarker of vasculitis and/or vascular leak in IBD, which may help to guide initial anti-TNF dosing until therapeutic drug monitoring can occur, warranting future study.

With therapy, the increase in mean ERL density in the longitudinal CD cohort correlated with the change in PCDAI, suggesting that NFC is also potentially useful in the monitoring of treatment response in patients with newly diagnosed CD. This supports reversibility of the microvascular effects of untreated IBD as evidenced by the increase in capillary number within the nailbed capillaries. Further investigation is still required to understand how quickly ERL density returns to normal values after starting therapy, if this can be expected at all, and how it correlates with mucosal healing or progression to stricturing or penetrating phenotypes.

The strengths of the study include the prospective, longitudinal nature of the study design, with new IBD patients reassessed up to 6 months after diagnosis. To date, this is the only study where NFC has been used both to distinguish between the pediatric IBD population and healthy controls and to monitor ERL changes in response to treatment in newly diagnosed patients. Similar to a prospective evaluation by Herrick et al, we did not find a difference in the ERL density with regard to age or gender.^16^ This identifies NFC as a potential screening and surveillance monitoring tool in IBD, warranting further prospective studies.

There are several limitations to this study. First, the study population is small, which may limit generalizability; however, these pilot findings support a need for additional examination of NFC and vasculitis in IBD. Second, the disease severity within the IBD population varied and included patients with active disease, patients in clinical remission for less than 6 months, and patients in clinical remission for longer than 6 months. It may be possible with a larger study size to separate cohort patients by disease states, particularly when evaluating the correlation between biomarkers of disease activity and mean ERL density. This concept is supported by our longitudinal data showing improvement in ERL density with therapy. Additionally, alternative quantitative measures of mucosal disease such as calprotectin were not performed regularly in this cohort. Given the young age of our patients, risk factors for nailfold changes including smoking or anticoagulation should be relatively low; however, we did not collect data on handedness, hobbies, or habits that could potentially affect the nailfold capillaries. Finally, we were unable to repeat NFC at consistent intervals after initial recruitment among the patients in our longitudinal CD population and unable to recruit a longitudinal UC cohort. This was due in part to irregular patient follow-up periods and varying durations of inpatient hospitalization.

## Conclusion

With the rising incidence of pediatric IBD comes the need for more accurate and noninvasive methods for disease monitoring. Current methods can be invasive, traumatic, costly, and time-consuming with delayed notification of test results. This is the first study that demonstrates that NFC can be used to document distinguishing nailfold patterns in pediatric IBD compared to healthy controls. With more frequent and widespread utilization of NFC in the office setting, it is possible that this method can be used as an adjunct in disease monitoring and tailoring of therapy. Further investigation is required to assess the relationship between ERL density and disease activity, possibly through correlation of ERL density with mucosal histologic findings obtained at the time of endoscopy.

## Data Availability

The data are not publicly available.
